# Akt (protein kinase B) isoform phosphorylation and signaling downstream of mTOR (mammalian target of rapamycin) in denervated atrophic and hypertrophic mouse skeletal muscle

**DOI:** 10.1186/1750-2187-7-7

**Published:** 2012-06-01

**Authors:** Marlene Norrby, Kim Evertsson, Ann-Kristin Fjällström, Anna Svensson, Sven Tågerud

**Affiliations:** 1 School of Natural Sciences, Linnaeus University, SE-391 82, Kalmar, Sweden; 2 Department of Clinical Sciences, Swedish University of Agricultural Sciences, SE-756 51, Uppsala, Sweden

**Keywords:** Akt, GSK-3β, 4EBP1, p70S6K1, rpS6, Denervation, Skeletal muscle, Mouse, Phosphorylation

## Abstract

**Background:**

The present study examines the hypothesis that Akt (protein kinase B)/mTOR (mammalian target of rapamycin) signaling is increased in hypertrophic and decreased in atrophic denervated muscle. Protein expression and phosphorylation of Akt1, Akt2, glycogen synthase kinase-3beta (GSK-3beta), eukaryotic initiation factor 4E binding protein 1 (4EBP1), 70 kD ribosomal protein S6 kinase (p70S6K1) and ribosomal protein S6 (rpS6) were examined in six-days denervated mouse anterior tibial (atrophic) and hemidiaphragm (hypertrophic) muscles.

**Results:**

In denervated hypertrophic muscle expression of total Akt1, Akt2, GSK-3beta, p70S6K1 and rpS6 proteins increased 2–10 fold whereas total 4EBP1 protein remained unaltered. In denervated atrophic muscle Akt1 and Akt2 total protein increased 2–16 fold. A small increase in expression of total rpS6 protein was also observed with no apparent changes in levels of total GSK-3beta, 4EBP1 or p70S6K1 proteins. The level of phosphorylated proteins increased 3–13 fold for all the proteins in hypertrophic denervated muscle. No significant changes in phosphorylated Akt1 or GSK-3beta were detected in atrophic denervated muscle. The phosphorylation levels of Akt2, 4EBP1, p70S6K1 and rpS6 were increased 2–18 fold in atrophic denervated muscle.

**Conclusions:**

The results are consistent with increased Akt/mTOR signaling in hypertrophic skeletal muscle. Decreased levels of phosphorylated Akt (S473/S474) were not observed in denervated atrophic muscle and results downstream of mTOR indicate increased protein synthesis in denervated atrophic anterior tibial muscle as well as in denervated hypertrophic hemidiaphragm muscle. Increased protein degradation, rather than decreased protein synthesis, is likely to be responsible for the loss of muscle mass in denervated atrophic muscles.

## Background

Maintenance of skeletal muscle mass is dependent upon a balance between anabolic and catabolic processes and signaling through the Akt (protein kinase B, PKB)/mTOR (mammalian target of rapamycin) pathway is believed to influence protein synthesis as well as protein degradation in skeletal muscle [1-3]. The Akt family consists of three different isoforms, Akt1, Akt2 and Akt3 (PKBα, β, γ) encoded by separate genes [4]. Gene deletion studies have indicated a role for both Akt1 and Akt2 in growth and skeletal muscle size [5] and overexpression of Akt1 has been shown to result in skeletal muscle hypertrophy [6]. Akt activity is regulated by phosphorylation both at a threonine site (T308 for Akt1) located in the central catalytic domain (see e.g. [4,7]) and at a serine site (S473 for Akt1) located in the C-terminal hydrophobic regulatory domain (see e.g. [4,8]). Phosphorylations of both sites are believed to be necessary for full activation of Akt kinase activity [9] although this may not be true for all Akt targets [10]. Akt has been implicated in the process of protein degradation based on its ability to phosphorylate Forkhead box O (Foxo) proteins (Foxo1, Foxo3a and Foxo4). Phosphorylation of Foxos results in sequestration in the cytoplasm thereby preventing Foxo-induced trans-cription of target genes, e.g. the ubiquitin ligases muscle-specific ring finger protein1 (MuRF1) and Atrogin1 (muscle atrophy F-box, MAFbx) ([11] see also e.g. [12]). Protein synthesis is influenced by Akt through at least two different mechanisms, including effects on glycogen synthase kinase-3β (GSK-3β) and on mTOR activity. GSK-3β is a direct substrate of Akt which by phosphory-lation of S9 inhibits GSK-3β mediated phosphorylation of eukaryotic initiation factor 2B (eIF2B) thereby activating eIF2B resulting in increased protein synthesis (see e.g. [13]). mTOR, on the other hand, is activated indirectly by Akt through phosphorylation of TSC2 in the TSC1/TSC2 (hamartin/tuberin) heterodimer that inhibits mTOR signaling (see [14]).

Increased signaling through mTOR is believed to enhance protein synthesis by increasing the translational capacity of the cell and by increasing the translation of certain mRNAs coding for translation factors (see e.g. [15,16]). The mTOR complex 1 (mTORC1), in which mTOR associates with raptor, is responsible for signaling to downstream substrates ([17] see also [18]). Raptor functions as a scaffolding protein for interactions between mTOR and the mTOR signaling (TOS) motif on downstream effector proteins (see [18]). Two substrates of mTOR that both contain TOS motifs are eukaryotic initiation factor 4E binding protein 1 (4EBP1) and 70 kD ribosomal protein S6 kinase (p70S6K1), that appear to work in parallel, yet distinct, pathways to control the size of mammalian cells ([19-22] see also [15,16]). Rapamycin-sensitive sites in p70S6K1 are the threonine sites T229, T389 and a serine site S404 with T389 appearing to be critical for kinase activity ([23,24] see also [25]). Phosphorylations of the substrate rpS6 occur in a distinct pattern with serine 236 (S236) being the first amino acid phosphorylated, followed by phosphorylation at S235, S240, S244 and finally S247 (see [26]). The second mTOR substrate, 4EBP1, acts as a repressor of translation initiation by binding to eukaryotic initiation factor 4E (eIF4E) thereby preventing the assembly of the translation initiation complex (eIF4F) commonly considered as the rate limiting step in translation (see e.g. [27,28]). Dissociation of the 4EBP1-eIF4E complex requires hyperphosphorylation of 4EBP1. Seven phosphorylation sites have been identified in 4EBP1 (T37, T46, S65, T70, S83, S101 and S112) and phosphorylation of the first four sites (T37, T46, S65, T70) are generally agreed to be of importance for the release of eIF4E (see e.g. [15]). These phosphorylations appear to be hierarchically regulated with phosphorylation first at T37 and T46 followed by T70 and lastly S65 [29,30].

Regarding models of skeletal muscle atrophy and hypertrophy the levels of S473 phosphorylated Akt is increased in models of skeletal muscle hypertrophy, such as functional overload of the rat or mouse plantaris muscle [1]. In atrophy models based on skeletal muscle inactivity, such as 10 days of hind-limb immobilization or 10–14 days of hind-limb suspension, Akt S473 phosphorylation has been reported to be decreased in rat medial gastrocnemius muscle [1] and soleus muscle [31-34] but not in rat extensor digitorum longus muscle [33]. In denervated skeletal muscle constitutively active Akt has been shown to inhibit atrophy of anterior tibial and soleus muscles [1,35] but little information has been published regarding the levels of different Akt isoforms or the levels of phosphorylated Akt in muscle denervated more than 1–3 days [36-40]. Inhibition of mTOR with rapamycin has been shown to prevent skeletal muscle hypertrophy [1] and mice with targeted disruption of the S6K1 gene display skeletal muscle atrophy [41]. Mouse embryonic fibroblasts (MEFs) deficient in the p70S6K1 substrate ribosomal protein S6 (rpS6) are significantly smaller than controls (see [42]) and increased phosphorylation of rpS6 has been demonstrated in skeletal muscle hypertrophy caused by synergist ablation [43-45] whereas decreased phosphorylation occurs in skeletal muscle atrophy caused by hind-limb unloading [44]. Mice deficient in rpS6 phosphorylation have decreased muscle mass and decreased abundance of contractile proteins [46].

The present study examines the hypothesis that the activities of Akt (as measured by S473/S474 phosphorylation of Akt1/Akt2) and mTOR (as measured by phosphory-lation of downstream substrates) are increased in hypertrophic muscle and decreased in atrophic muscle using a model of denervated skeletal muscles. Thus, the protein expression and phosphorylation status of Akt1 (S473), Akt2 (S474), GSK-3β (S9), 4EBP1 (S65), p70S6K1 (T389) and rpS6 (S235/236) were examined in innervated and 6-days denervated hemidiaphragm muscles and in innervated and 6-days denervated anterior tibial muscles from mice. The hemidiaphragm muscle becomes transiently hypertrophic following denervation [47-49] whereas the anterior tibial muscle, like most other skeletal muscles, undergoes continuous atrophy following denervation. The transient hypertrophy of the denervated hemidiaphragm may be due to passive stretching caused by continued contractions in the contralateral innervated hemidiaphragm since bilateral denervation does not induce hypertrophy [47-49]. The transient hypertrophy of the hemidiaphragm lasts 6–10 days after which the muscle decreases in weight and gradually becomes atrophic [48,49]. The results are consistent with a role for Akt and mTOR activation in hypertrophic denervated hemidiaphragm. Evidence of increased mTOR signaling and absence of decreased Akt activation (S473/S474 phosphorylation) in atrophic denervated anterior tibial muscle suggests that other signaling mechanisms are responsible for the atrophic process in this denervated muscle.

## Results

### Muscle weights

**Figure 1:**
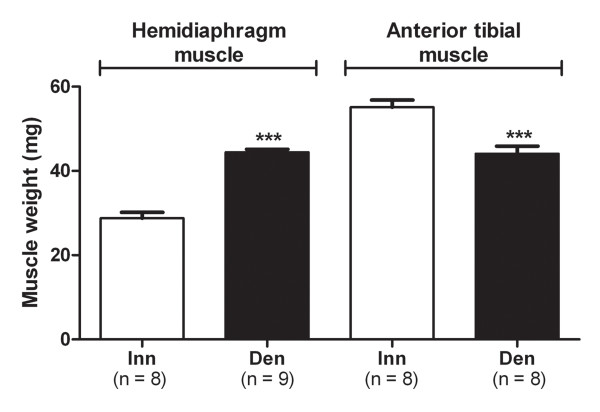
**Muscle weights.** Muscle weights of 6-days denervated (Den) hypertrophic hemidiaphragm muscles and 6-days denervated atrophic anterior tibial muscles compared to innervated (Inn) controls. Mean values ± standard error of the mean. ***p < 0.001.

Six days after denervation hemidiaphragm muscles were hypertrophic with a wet weight of 44.4 ± 0.7 mg (n = 9) compared to 28.8 ± 1.4 mg (n = 8) for innervated controls (p < 0.001, Student’s t-test, Figure 1). Six days after denervation anterior tibial muscles were atrophic with a wet weight of 44.1 ± 1.8 mg (n = 8) compared to 55.1 ± 1.7 mg (n = 8) for innervated controls (p < 0.001, Student’s t-test, Figure 1).

### Protein expression in hypertrophic 6-days denervated hemidiaphragm muscle

**Figure 2:**
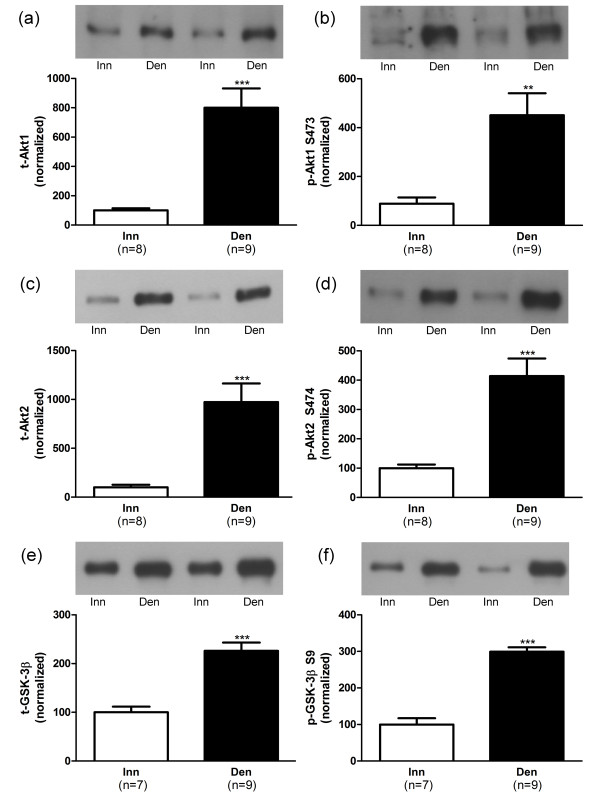
**Akt and GSK-3β protein and phosphorylation levels in 6-days denervated hypertrophic hemidiaphragm muscle.** Expression of Akt1, Akt2 and GSK-3β total protein (t-Akt1, a; t-Akt2, c and t-GSK-3β, e) and phosphorylated Akt1 protein (p-Akt1) at S473 (b), phosphorylated Akt2 protein (p-Akt2) at S474 (d) and phosphorylated GSK-3β protein (p-GSK-3β) at S9 (f) in 6-days denervated hypertrophic hemidiaphragm muscle (Den) compared to innervated (Inn) controls. Representative Western blots are shown together with densitometric quantifications. One innervated hemidiaphragm muscle sample was used as a reference sample and was included in all gels. All other samples were measured relative to this reference. The data were normalized to give an average signal of 100.0 in innervated muscles. Mean values ± standard error of the mean. **p < 0.01, ***p < 0.001.

**Figure 3:**
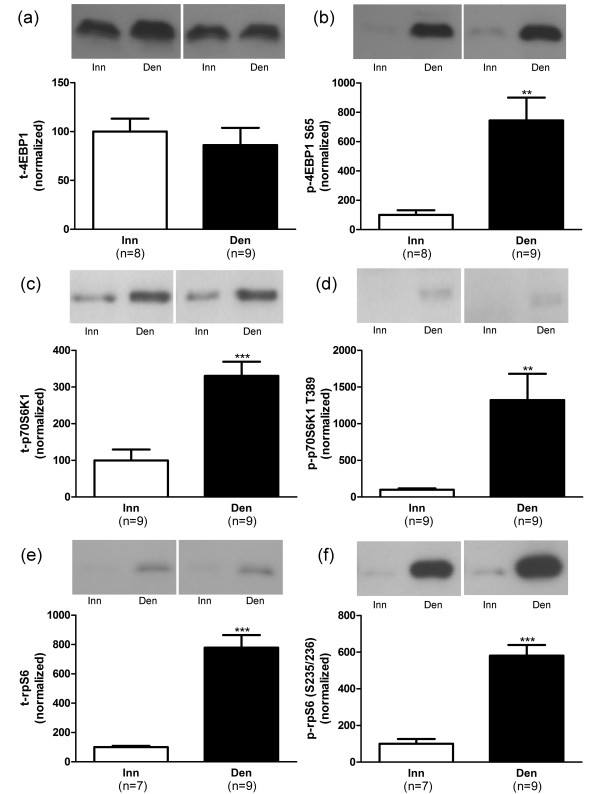
**4EBP1, p70S6K1 and rpS6 protein and phosphorylation levels in 6-days denervated hypertrophic hemidiaphragm muscle.** Expression of 4EBP1, p70S6K1 and rpS6 total protein (t-4EBP1, a; t-p70S6K1, c and t-rpS6, e) and phosphorylated 4EBP1 (p-4EBP1) at S65 (b), phosphorylated p70S6K1 (p-p70S6K1) at T389 (d) and phosphorylated rpS6 (p-rpS6) at S235/236 (f) in 6-days denervated hypertrophic hemidiaphragm muscle (Den) compared to innervated (Inn) controls. Representative Western blots are shown together with densitometric quantifications. One innervated hemidiaphragm muscle sample was used as a reference sample and included in all gels. All other samples were measured relative to this reference. The data were normalized to give an average signal of 100.0 in innervated muscles. Mean values ± standard error of the mean. **p < 0.01, ***p < 0.001.

In hypertrophic 6-days denervated hemidiaphragm muscles the mean expression of total Akt1, Akt2 and GSK-3β proteins as well as the expression of phosphorylated Akt1 (S473), Akt2 (S474) and GSK-3β (S9) were significantly up-regulated (Figure 2). The mean expression of total p70S6K1 and rpS6 proteins were also significantly increased whereas no such change was observed for the expression of total 4EBP1. The mean expression of phosphorylated 4EBP1 (S65), p70S6K1 (T389) and rpS6 (S235/236) were significantly increased compared to innervated controls (Figure 3).

The mean expression level of total Akt1 protein (Figure 2a) was 800.8 ± 131.6 arbitrary units (n = 9) in denervated muscles compared to 100.0 ± 14.9 (n = 8) in innervated muscles (p < 0.001, Student’s t-test). The mean expression level of total Akt2 was 972.5 ± 190.8 arbitrary units (n = 9) in denervated muscles compared to 100.0 ± 26.5 (n = 8) in innervated muscles (p < 0.001, Student’s t-test, Figure 2c). The mean expression level of total GSK-3β was 226.1 ± 17.0 arbitrary units (n = 9) in denervated muscles compared to 100.0 ± 11.7 (n = 7) in innervated muscles (p < 0.001, Student’s t-test, Figure 2e).

The mean expression level of Akt1 protein phosphorylated at S473 (Figure 2b) was 450.7 ± 90.0 arbitrary units (n = 9) in denervated muscles compared to 100.0 ± 26.0 (n = 8) in innervated muscles (p < 0.01, Student’s t-test). The mean expression level of phosphorylated Akt2 (S474) was 414.0 ± 59.8 arbitrary units (n = 9) in denervated muscles compared to 100.0 ± 12.4 (n = 8) in innervated muscles (p < 0.001, Student’s t-test, Figure 2d). The mean expression level of phosphorylated GSK-3β (S9) was 299.2 ± 12.0 arbitrary units (n = 9) in denervated muscles compared to 100.0 ± 17.0 (n = 7) in innervated muscles (p < 0.001, Student’s t-test, Figure 2f).

The mean expression level of total 4EBP1 protein was 86.1 ± 17.6 arbitrary units (n = 9) in denervated muscles compared to 100.0 ± 13.2 (n = 8) in innervated muscles (Figure 3a). The mean expression level of total p70S6K1 protein was 330.5 ± 38.7 arbitrary units (n = 9) in denervated muscles compared to 100.0 ± 29.7 (n = 9) in innervated muscles (p < 0.001, Student’s t-test, Figure 3c). The mean expression level of total rpS6 protein was 779.0 ± 85.6 arbitrary units (n = 9) in denervated muscles compared to 100.0 ± 9.1 (n = 7) in innervated muscles (p < 0.001, Student’s t-test, Figure 3e).

The mean expression level of phosphorylated 4EBP1 (S65) was 744.2 ± 156.0 arbitrary units (n = 9) in denervated muscles compared to 100.0 ± 32.0 (n = 8) in innervated muscles (p < 0.01, Student’s t-test, Figure 3b). The mean expression level of phosphorylated p70S6K1 (T389) was 1322 ± 358 arbitrary units (n = 9) in denervated muscles compared to 100.0 ± 17.8 (n = 9) in innervated muscles (p < 0.01, Student’s t-test, Figure 3d). The mean expression level of phosphorylated rpS6 (S235/236) was 580.7 ± 58.3 arbitrary units (n = 9) in denervated muscles compared to 100.0 ± 26.3 (n = 7) in innervated muscles (p < 0.001, Student’s t-test, Figure 3f).

### Protein expression in atrophic 6-days denervated anterior tibial muscle

**Figure 4:**
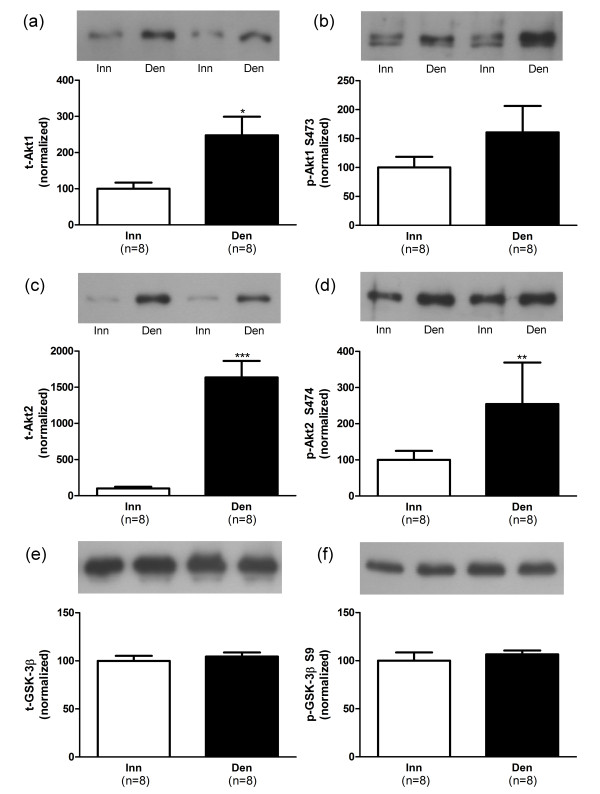
**Akt and GSK-3β protein and phosphorylation levels in 6-days denervated atrophic anterior tibial muscle**. Expression of Akt1, Akt2 and GSK-3β total protein (t-Akt1, a; t-Akt2, c and t-GSK-3β, e) and phosphorylated Akt1 protein (p-Akt1) at S473 (b), phosphorylated Akt2 protein (p-Akt2) at S474 (d) and phosphorylated GSK-3β protein (p-GSK-3β) at S9 (f) in 6-days denervated atrophic anterior tibial (Den) muscle compared to innervated (Inn) controls. Representative Western blots are shown together with densitometric quantifications. One innervated anterior tibial muscle sample was used as a reference sample and was included in all gels. All other samples were measured relative to this reference. The data were normalized to give an average signal of 100.0 in innervated muscles. Mean values ± standard error of the mean. *p < 0.05, **p < 0.01, ***p < 0.001.

**Figure 5:**
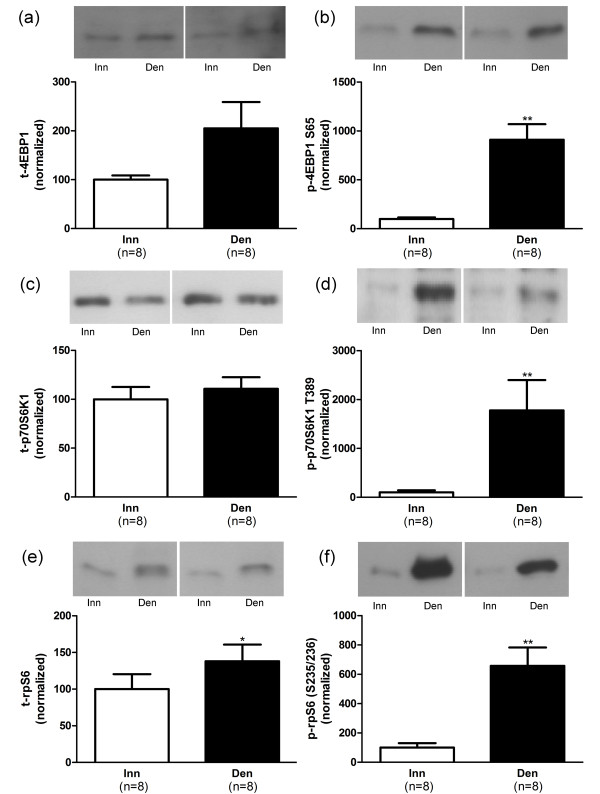
**4EBP1, p70S6K1 and rpS6 protein and phosphorylation levels in 6-days denervated atrophic anterior tibial muscle.** Expression of 4EBP1, p70S6K1 and rpS6 total protein (t-4EBP1, a; t-p70S6K1, c and t-rpS6, e) and phosphorylated 4EBP1 (p-4EBP1) at S65 (b), phosphorylated p70S6K1 (p-p70S6K1) at T389 (d) and phosphorylated rpS6 (p-rpS6) at S235/236 (f) in 6-days denervated atrophic anterior tibial muscle (Den) compared to innervated (Inn) controls. Representative Western blots are shown together with densitometric quantifications. One innervated anterior tibial muscle sample was used as a reference sample and included in all gels. All other samples were measured relative to this reference. The data were normalized to give an average signal of 100.0 in innervated muscles. Mean values ± standard error of the mean. *p < 0.05, **p < 0.01.

In atrophic 6-days denervated anterior tibial muscles the mean expression of total Akt1 and Akt2 proteins were significantly up-regulated whereas no significant alteration in total GSK-3β expression was observed compared to innervated controls (Figure 4). The mean expression of phosphorylated Akt2 (S474) was significantly up-regulated but the mean expression of phosphorylated Akt1 (S473) and phosphorylated GSK-3β (S9) were not significantly different from innervated control muscles (Figure 4). The mean expression of total 4EBP1 and p70S6K1 proteins were not significantly altered compared to innervated controls but a small, statistically significant, increase in expression of total rpS6 protein was observed (Figure 5). The levels of phosphorylated 4EBP1 (S65), p70S6K1 (T389) and rpS6 (S235/236) were significantly increased in denervated atrophic anterior tibial muscles compared to innervated controls (Figure 5).

The mean expression level of total Akt1 protein (Figure 4a) was 248.0 ± 51.2 arbitrary units (n = 8) in denervated muscles compared to 100.0 ± 16.9 (n = 8) in innervated muscles (p < 0.05, Student’s paired *t*-test). The mean expression level of total Akt2 was 1636 ± 228 arbitrary units (n = 8) in denervated muscles compared to 100.0 ± 24.1 (n = 8) in innervated muscles (p < 0.001, Student’s paired *t*-test, Figure 4c). The mean expression level of total GSK-3β protein was 104.5 ± 4.2 arbitrary units (n = 8) in denervated muscles compared to 100.0 ± 5.2 (n = 8) in innervated muscles (Figure 4e).

The mean expression level of Akt1 protein phosphorylated at S473 (Figure 4b) was 160.8 ± 45.5 arbitrary units (n = 8) in denervated muscles compared to 100.0 ± 18.5 (n = 8) in innervated muscles. The mean expression level of phosphorylated Akt2 (S474) was 254.5 ± 114.5 arbitrary units (n = 8) in denervated muscles compared to 100.0 ± 24.7 (n = 8) in innervated muscles (p < 0.01, Wilcoxon matched pairs test, Figure 4d). The mean expression level of phosphorylated GSK-3β (S9) was 106.6 ± 4.0 arbitrary units (n = 8) in denervated muscles compared to 100.0 ± 8.6 (n = 8) in innervated muscles (Figure 4f).

The mean expression level of total 4EBP1 protein was 205.0 ± 53.8 arbitrary units (n = 8) in denervated muscles compared to 100.0 ± 8.6 (n = 8) in innervated muscles (Figure 5a). The mean expression level of total p70S6K1 protein was 110.7 ± 11.8 arbitrary units (n = 8) in denervated muscles compared to 100.0 ± 12.6 (n = 8) in innervated muscles (Figure 5c). The mean expression level of total rpS6 protein was 138.1 ± 22.7 arbitrary units (n = 8) in denervated muscles compared to 100.0 ± 20.4 (n = 8) in innervated muscles (p < 0.05, Student’s paired t-test, Figure 5e).

The mean expression level of phosphorylated 4EBP1 (S65) was 910.6 ± 158.5 arbitrary units (n = 8) in denervated muscles compared to 100.0 ± 16.1 (n = 8) in innervated muscles (p < 0.01, Student’s paired t-test, Figure 5b). The mean expression level of phosphorylated p70S6K1 (T389) was 1778 ± 622 arbitrary units (n = 8) in denervated muscles compared to 100.0 ± 42.6 (n = 8) in innervated muscles (p < 0.01, Wilcoxon matched pairs signed rank test, Figure 5d). The mean expression level of phosphorylated rpS6 (S235/236) was 657.5 ± 125.6 arbitrary units (n = 8) in denervated muscles compared to 100.0 ± 30.6 (n = 8) in innervated muscles (p < 0.01, Student’s paired t-test, Figure 5f).

### Akt mRNA expression in atrophic 6-days denervated hind-limb muscles

**Figure 6:**
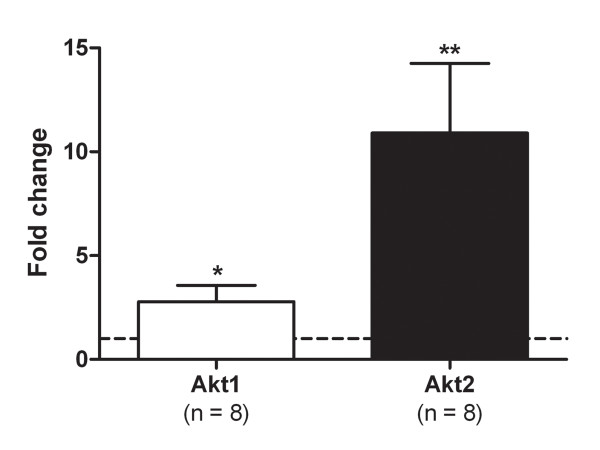
**Akt mRNA expression.** Akt1 and Akt2 mRNA expression in 6-days denervated atrophic hind-limb muscles (pooled muscle samples containing anterior tibial, extensor digitorum longus, soleus and gastrocnemius muscles) compared to innervated controls and expressed as fold change. Mean values ± standard error of the mean. *p < 0.05, **p < 0.01. The dotted line represents a fold change of 1, equal to no difference in expression between denervated and innervated muscles.

The mRNA expressions of both Akt1 and Akt2 were significantly up-regulated in 6-days denervated atrophic muscles compared to innervated controls (Figure 6) with fold changes of 2.78 ± 0.79 for Akt1 (n = 8, p < 0.05, compared to the hypothetical value 1.00, Wilcoxon signed rank test) and 10.91 ± 3.35 for Akt2 (n = 8, p < 0.01, compared to the hypothetical value 1.00, Wilcoxon signed rank test).

## Discussion

The Akt/mTOR signaling pathway is proposed to play a major role in the regulation of skeletal muscle mass ([50] see also [3]). In the present study the expression levels and phosphorylation status of Akt isoforms, the Akt substrate GSK-3β and of proteins located downstream of mTOR were examined in a model of skeletal muscle hypertrophy and atrophy consisting of 6-days denervated hemidiaphragm muscle (hypertrophic) and 6-days denervated anterior tibial muscle (atrophic). The hemidiaphragm muscle becomes transiently hypertrophic following denervation [47-49,51] whereas the anterior tibial muscle, like most other adult skeletal muscles, undergoes continuous atrophy following denervation (e.g. [51,52]).

The results of the present study are consistent with a number of previous studies indicating increased signaling through the Akt/mTOR pathway in hypertrophic skeletal muscle [1,50,53]. Increased expression of phosphorylated p70S6K1 and 4EBP1 proteins in denervated rat hemidiaphragm muscle has been reported previously [54] as has increased rpS6 phosphorylation [55] and increased phosphorylation of GSK-3β [56].

In contrast to some previous studies, on other models of skeletal muscle atrophy, no evidence of decreased signaling through the Akt/mTOR pathway was observed in atrophic denervated anterior tibial muscle in the present study. Similarly, no decreased phosphorylation of GSK-3β (S9) was observed in atrophic denervated anterior tibial muscle as also reported previously [56]. Increased levels of Akt total protein and phosphorylated Akt were also recently reported in atrophic mouse muscles 2 weeks following denervation [37]. These results suggest that signaling mechanisms other than decreased Akt activity/phosphorylation are responsible for the atrophic process in at least some denervated muscles. Previous studies on immobilized human muscle have also indicated decreased responsiveness of muscle protein synthesis to amino acids without any clear evidence of decreased Akt signaling [57]). A difference in the mechanisms responsible for muscle loss following denervation and hind-limb suspension has been suggested previously [58]. A suppression of protein synthesis was suggested to possibly be more important after hind-limb suspension whereas increased protein degradation may be more important after denervation [58].

It is interesting to note that the large increases observed in phosphorylated 4EBP1, p70S6K1 and rpS6 are similar in atrophic and hypertrophic denervated muscles whereas substantial increases in total proteins were only observed for p70S6K1 and rpS6 in denervated hypertrophic muscle. Since the exact roles of p70S6K1 and rpS6 in the regulation of protein synthesis are not entirely clear (see [26,42]) it is not obvious how these differences should be interpreted. Furthermore, changes in the phosphorylation status of signaling molecules such as p70S6K1 and 4EBP1 may not always imply changes in protein synthesis [59,60]. Nevertheless, the present results may well be in line with previous studies indicating increased protein synthesis in hypertrophic hemidiaphragm muscle [61] as well as in atrophic hind-limb muscles of adult mice [52] denervated for similar time periods as in the present study. Increased phosphorylation of p70S6K1 in extensor digitorum longus muscles denervated for 7 days and in gastrocnemius and soleus muscles denervated for two weeks has also been reported previously [37,62].

Increased protein synthesis in denervated skeletal muscle may, at least in part, be related to denervation changes other than those causing alterations in muscle mass. Following denervation a number of proteins are differentially expressed in denervated compared to innervated muscles. Thus, the expression of myosin isoforms changes following denervation ([63,64] see also [65]) and acetylcholine receptors are expressed in extrasynaptic areas of the sarcolemma [66,67]. Many properties of denervated muscle, such as the increased expression of acetylcholine receptors, expression of the embryonic acetylcholine receptor gamma-subunit [68], expression of tetrodotoxin-resistant sodium channels ([69,70] see also [65]) and increased expression of myogenic factors, such as myogenin and MyoD [71-73], resemble properties of developing muscles. Myogenin was recently suggested to control denervation-dependent skeletal muscle atrophy [74,75] and MyoD has been proposed to participate in a positive feedback regulation with Akt2 in muscle differentiation [76], a process in which Akt 1 has also been implicated [77]. Increased expression of ribosomes [66] and anabolic factors such as Akt and rpS6 (as seen in the present study) may thus be related to increased synthesis of specific proteins required for the tissue remodelling that occurs after denervation.

Taken together, the results of the present study and those of a number of previous studies indicate that skeletal muscle atrophy following denervation is more likely to depend on increased protein degradation than on an overall decrease in protein synthesis. Ubiquitin-proteasome-dependent proteolysis appears to play a major role in muscle protein degradation including increased proteolysis following denervation [78-81]. The E3-ubiquitin ligases muscle ring finger protein 1 (MuRF1) and muscle atrophy F-box (MAFbx, Atrogin1) appear to be critical and their mRNA expression levels increase in a number of different atrophic conditions including denervation [82-88]. Increased Akt activity has been suggested to decrease the expression of MuRF1 and MAFbx based on the ability of Akt to phosphorylate Foxo transcription factors [11,89]. In atrophic denervated muscle, however, unchanged or increased Akt phosphorylation/activity suggests that other signaling mechanisms are of importance for regulating the expression of MuRF1 and MAFbx in denervated muscle. Such signaling mechanisms may include tumor necrosis factor-like (TNF-like) weak inducer of apoptosis (TWEAK) and its receptor, fibroblast growth factor-inducible receptor 14 (Fn14) [38], nuclear factor-kappaB (NF-κB [39,90], the JunB transcription factor [91], myogenin [74,75], mitogen-activated protein kinase 14 (p38 MAPK) [92], heat shock proteins such as Hsp70 and Hsp25rodent/27human [93-95] and mitogen-activated protein kinase-activated protein kinase 2 (MK2 or MAPKAPK2), a substrate of p38 MAPK [51].

## Conclusions

This study has examined the hypothesis that Akt/mTOR signaling is increased in hypertrophic muscle and decreased in atrophic muscle using a model consisting of different denervated skeletal muscles. The results are consistent with previous studies showing increased Akt/mTOR signaling in models of hypertrophic skeletal muscle. In contrast to previous studies, on other models of skeletal muscle atrophy, the present study found no evidence of decreased Akt phosphorylation (S473/S474) in atrophic denervated anterior tibial muscle. The results of the present study also suggest increased signaling through mTOR, indicating increased protein synthesis, in denervated atrophic muscles as well as in denervated hypertrophic muscles. Increased protein degradation, rather than decreased protein synthesis, is therefore likely to be responsible for the loss of muscle mass in denervated atrophic muscles. Some of the alterations in protein expression and phosphorylation observed in the present study may be related to denervation changes in skeletal muscle other than those directly involved in the regulation of muscle mass.

## Methods

### Animals

All experiments were performed on adult male NMRI mice (NOVA-SCB, Sollentuna, Sweden). Before surgery the animals were anaesthetized by inhalation of isoflurane or sevoflurane. Denervation of the left hind-limb or the left hemidiaphragm was performed by sectioning and removing a few mm of the sciatic nerve or phrenic nerve as described previously [96]. While still anaesthetized animals received a subcutaneous injection of buprenorphine (50 μg/kg) for analgesia. Six days after denervation mice were killed by cervical dislocation. The experimental manipulations have been approved by the Ethical Committee for Animal Experiments, Linköping, Sweden.

### Protein extraction

Mouse hemidiaphragm and anterior tibial muscles were used for protein extraction. Following dissection and weighing the muscles were frozen on dry ice and stored at −80°C. The muscles were later homogenized in 1 ml of a buffer containing 100 mM Tris-HCl, pH 7.6, 150 mM NaCl, 1 mM EDTA, 1% NP-40, 0.1% sodium deoxycholate, 2 mM Na_3_VO_4_ and 100 mM NaF with one Protease Inhibitor Cocktail Mini Tablet (Roche Diagnostics GmbH, Mannheim, Germany) per 10 ml of extraction buffer and centrifuged. The supernatant was recovered and the pellet was resuspended in 0.5 ml of buffer and recentrifuged. The supernatants were combined and the protein concentration was determined as described in [97].

### Western blots

Western blots were prepared essentially as described in [97]. Five to forty μg protein were reduced, denatured and electrophoretically separated on a 12% polyacrylamide gel with a 5.2% polyacrylamide stacking gel on top. Gels were electroblotted onto PVDF Plus transfer membranes (GE Water & Process Technologies, Trevose, PA, U.S.A. or Amersham Hybond-P, GE Healthcare, Buckinghamshire, England) and the membranes were blocked and then incubated with antibodies. Primary antibodies for detecting total Akt1 [2967], total Akt2 [2962], phospho-GSK-3β (S9) [9336], total 4EBP1 [9452], phospho-4EBP1 (S65) [9451], total S6K1 [9202], phospho-S6K1 (T389) [9205], total rpS6 [2317] and phospho-rpS6 (S235/236) [2211] were from Cell Signaling Technology (Beverly, CA). Primary antibody for detecting phospho-Akt1 (S473) [07-310] was from Upstate Cell Signaling Solutions (Lake Placid, NY), primary antibody for detecting phospho-Akt2 (S474) [ab38513] was from Abcam (Cambridge, UK) and primary antibody for detecting total GSK-3β [610202] was from BD Transduction Laboratories (San Diego, CA). All primary antibodies were used at a dilution of 1/500 – 1/2000. Antibodies were visualized with horseradish peroxidase conjugated secondary immunoglobulin diluted 1/1000 – 1/10000 (goat anti-rabbit IgG [P0448] or rabbit anti-mouse [P0260] Dako, Glostrup, Denmark). Negative controls included membranes incubated in the absence of the primary antibodies. The bound immune complexes were detected using the ECL Plus Western blotting detection system and Hyperfilm ECL (Amersham International and Amersham Pharmacia Biotech, Buckinghamshire, England).

### RNA extraction

For RNA extraction gastrocnemius, soleus, anterior tibial and extensor digitorum longus muscles from 6-days denervated hind-limbs were dissected, pooled and then processed together for RNA extraction. The same muscles from the contralateral leg were pooled separately and used as innervated controls. RNA was extracted as described in [96].

### Quantitative real-time PCR

Real-time PCR analysis was performed essentially as described in [98] using cDNA reverse transcribed from 1 μg of total RNA extracted from 6-days denervated and innervated hind-limb muscles. The primers used were for Akt1 (sequences 5′ to 3′) GCCTACCGAGAAGAXGACTCTGA and GTCTTCATCAGCTGGCATTGT, and were designed to amplify a 260 bp cDNA fragment corresponding to nucleotides 218-477 of the mouse Akt1 mRNA sequence [GenBank: X65687, [99]]. Primers for Akt2 were TAAAAAGTGGCTCTGGTGTGTG and GGCATTCTGCTACAGAGAAATTG, and were designed to amplify a 331 bp cDNA fragment corresponding to nucleotides 37-367 of the mouse Akt2 mRNA sequence [GenBank: NM_007434.2, [100]]. Each cDNA was analyzed in triplicates by real-time PCR reactions using the Applied Biosystems 7500 Real-Time PCR system (Applied Biosystems, Stockholm, Sweden) and C_t_ (threshold cycle) values were determined with ABI seq detection software version 1.3.1. Mean C_t_-values for paired innervated and denervated samples were subtracted to give ΔC_t_ values and these were then converted to fold change in expression for denervated compared to innervated muscles (2^ΔCt^). The data obtained were not related to any internal control gene.

### Data analysis and statistics

The expression levels of total and phosphorylated proteins were studied semi-quantitatively using data from the Western blots. Equal amounts of total protein from innervated and denervated muscles were loaded on the gels. Measured levels of total and phosphorylated proteins were expressed without normalization to any specific protein. No “loading controls” were used and any differences in protein quantifications, pipetting steps, protein transfers etc. are included in the variations of the data sets.

Image analysis was performed using the gel plotting macro of the program ImageJ (Rasband, W.S., ImageJ, US National Institutes of Health, Bethesda, Maryland, USA, http://rsb.info.nih.gov/ij/, 1997–2007). Results were obtained in uncalibrated optical density units.

For quantification of protein expression one of the innervated anterior tibial muscle samples was used as a reference sample and was included in all gels. All other samples were measured relative to this reference, the signal of which was set to 100.0. Hemidiaphragm muscle samples were analyzed in a similar manner using one innervated sample as a reference sample (signal value 100.0) against which all other samples were measured. In the final analysis all signals were, again, normalized in such a way that the average signal from innervated muscles became 100.0.

Data are presented as mean values ± standard error of the mean (SEM). Student’s t-test was used for statistical comparisons of normally distributed data (according to D’Agostino-Pearson omnibus K2 normality test). Statistical significance for data not being normally distributed was determined using the Mann-Whitney test (hemidiaphragm muscles). The Wilcoxon signed rank test was used for comparing fold changes in mRNA expression to the hypothetical value 1.00. Mean expression in denervated muscle was considered as significantly different from that in innervated muscle if p < 0.05.

## Misc

Kim Evertsson and Ann-Kristin Fjällström contributed equally to this work.

## Competing interests

The authors declare no competing interests.

## Authors’ contributions

The work presented here was carried out in collaboration between all authors. All authors were involved in the design of the study. MN, KE and AKF carried out most of the protein expression studies, statistical analyses and wrote drafts of the manuscript. MN supervised most of the experimental work. ST conceived of the study, participated in the statistical analyses and finalized the manuscript. AS critically read and commented the manuscript. All authors have read and approved the final manuscript.
